# Metabolic labeling in middle-down proteomics allows for investigation of the dynamics of the histone code

**DOI:** 10.1186/s13072-017-0139-z

**Published:** 2017-07-06

**Authors:** Simone Sidoli, Congcong Lu, Mariel Coradin, Xiaoshi Wang, Kelly R. Karch, Chrystian Ruminowicz, Benjamin A. Garcia

**Affiliations:** 1 Department of Biochemistry and Biophysics, Epigenetics Institute, Perelman School of Medicine, University of Pennsylvania, Room 9-124, 3400 Civic Center Blvd, Bldg 421, Philadelphia, PA 19104 USA; 2 Białystok, Poland

**Keywords:** Epigenetics, Histones, Mass spectrometry, Methylation, Middle-down, Posttranslational modifications, SILAC

## Abstract

**Background:**

Middle-down mass spectrometry (MS), i.e., analysis of long (~50–60 aa) polypeptides, has become the method with the highest throughput and accuracy for the characterization of combinatorial histone posttranslational modifications (PTMs). The discovery of histone readers with multiple domains, and overall the cross talk of PTMs that decorate histone proteins, has revealed that histone marks have synergistic roles in modulating enzyme recruitment and subsequent chromatin activities. Here, we demonstrate that the middle-down MS strategy can be combined with metabolic labeling for enhanced quantification of histone proteins and their combinatorial PTMs in a dynamic manner.

**Methods:**

We used a nanoHPLC-MS/MS system consisting of hybrid weak cation exchange–hydrophilic interaction chromatography combined with high resolution MS and MS/MS with ETD fragmentation. After spectra identification, we filtered confident hits and quantified polypeptides using our in-house software isoScale.

**Results:**

We first verified that middle-down MS can discriminate and differentially quantify unlabeled from heavy labeled histone N-terminal tails (heavy lysine and arginine residues). Results revealed no bias toward identifying and quantifying unlabeled versus heavy labeled tails, even if the heavy labeled peptides presented the typical skewed isotopic pattern typical of long protein sequences that hardly get 100% labeling. Next, we plated epithelial cells into a media with heavy methionine-(methyl-^13^CD_3_), the precursor of the methyl donor *S*-adenosylmethionine and stimulated epithelial to mesenchymal transition (EMT). We assessed that results were reproducible across biological replicates and with data obtained using the more widely adopted bottom-up MS strategy, i.e., analysis of short tryptic peptides. We found remarkable differences in the incorporation rate of methylations in non-confluent cells versus confluent cells. Moreover, we showed that H3K27me3 was a critical player during the EMT process, as a consistent portion of histones modified as H3K27me2K36me2 in epithelial cells were converted into H3K27me3K36me2 in mesenchymal cells.

**Conclusions:**

We demonstrate that middle-down MS, despite being a more scarcely exploited MS technique than bottom-up, is a robust quantitative method for histone PTM characterization. In particular, middle-down MS combined with metabolic labeling is currently the only methodology available for investigating turnover of combinatorial histone PTMs in dynamic systems.

**Electronic supplementary material:**

The online version of this article (doi:10.1186/s13072-017-0139-z) contains supplementary material, which is available to authorized users.

## Background

 DNA and histone proteins are the major components of chromatin, a highly organized and dynamic macromolecular element in cell nuclei. Due to their intimate association with DNA, chromatin proteins modulate the transcription of genes, recruit enzymes responsible for DNA repair and also moderate condensation of DNA into chromosomes during mitosis and meiosis [1]. The basic unit of chromatin is the nucleosome, comprised of an assembly of eight histones, four histone types with two copies each, wrapped around by DNA every ~200 base pairs. Histones are heavily modified by posttranslational modifications (PTMs); such modifications contribute to all the above-mentioned chromatin functions mostly by serving as binding target for readers that get recruited on the chromatin [2]. For about 15 years, it has been proposed that histone sequence variations and combinations of PTMs compose a sort of “code” [3] that complements the genetic code in modulating chromatin activities. Nowadays the term “code” is not used as much anymore, mostly because it is yet unraveled. However, there are many examples of enzymes with multiple domains docking more than one histone PTM (e.g., [4]) and examples of cross talk between histone PTMs [5, 6]. For instance, both phosphorylation on serine 10 (S10ph) and acetylation on lysine 14 (K14ac) are required on the same histone H3 tail to activate the transcription of the gene p21, where only one of the two PTMs does not suffice [7]. As well, the binding of the heterochromatin protein 1 (HP1, or CBX) to the histone H3 mark K9me3 is obstructed and leads to protein release in the presence of H3S10ph [8]. Another example is that H3K4ac inhibits the binding of the protein spChp1 to H3K9me2/me3 in *S. pombe* [9]. Overall, these examples highlight the highly synergistic role of combinatorial PTMs and emphasize the need to quantify the total PTM content of a single histone tail.

Another open debate is whether histone PTMs are actually epigenetics, meaning that they are preserved during cell division, and which mechanisms are used for transgenerational inheritance (e.g., [10, 11]). Some studies propose that histone writers, and not histone PTMs, are preserved during replication (e.g., [12]). In general, understanding aberrant regulation of these heritable gene expression patterns is essential, as it is becoming increasingly evident that they are implicated in many disease pathologies, including cancer [13–15]. However, investigating this kind of histone PTM dynamics is not trivial, as it requires a methodology that can accurately quantify combinatorial histone PTMs, possibly discriminating old versus newly synthesized histones and their respective PTMs. Genetically encoded fluorescent reporters have been proposed as a suitable technique to understand PTM dynamics. This method involves tagging the protein of interest with two fluorophores using cloning techniques, and performing FRET analysis to determine the dynamics based on conformational changes [16, 17]. However, these experiments have several pitfalls to consider: (1) The protein crystal structure needs to be known to place the fluorophore in a key location that does not disturb the native secondary structure of the protein; (2) stable cell lines that express the desired constructs may not be obtained; (3) only one protein can be studied in a single experiment; and (4) no more than 2 modifications can be studied at same time. Modification-specific intracellular antibodies (mintbodies) were also developed to monitor the levels of histone PTMs in dynamic systems, thus overcoming some of the FRET-based techniques limitations [18]. This method provides information about the spatial distribution of a given mark, but only one PTM at a time can be studied in each experiment. Bernstein and co-workers also published a genomics approach to looking at combinatorial PTMs; the approach performs high-throughput single-molecule imaging and it can cope with multiple histone PTMs [19]. Overall, even though these methods have proved very successful in dedicated applications, they have all the limitations associated with the use of antibodies, including the fact that binding can be affected by nearby PTMs, which occurs frequently for hypermodified proteins such as histones.

Mass spectrometry (MS) is currently the ideal method to study histone PTMs [20], as high mass accuracy, speed and flexibility in MS/MS fragmentation allow for the rapid characterization of peptide sequences with unknown modifications [21]. MS has been widely applied in characterizing histones upon epigenetic aberrations, particularly related to abnormal levels of PTMs and histone mutations (reviewed in [22]). Middle-down MS is the sub-discipline of proteomics that adopts partial protein digestion to characterize coexisting PTMs. Usually, histones are purified and digested using GluC, because cleavage after glutamic acid cleaves the entire histone N-terminal tail from the nucleosome core. Most histone PTMs occur on the tails and so GluC digestion preserves the connectivity between the majority of histone PTMs [23]. After improvements in separation using a hybrid weak cation exchange–hydrophilic interaction chromatography (WCX–HILIC) [23], and efficient MS/MS fragmentation using electron transfer dissociation (ETD) [24], we optimized middle-down MS for histone tail analysis to a high-throughput platform [25]. In the same work, we introduced isoScale [25] as software tool to filter results from traditional proteomics search engines (not optimized for middle-down MS). isoScale also performs peptide quantification, including co-fragmented isobaric species, using the fragment ion relative ratio (FIRR) approach proposed by Pesavento et al. [26]. Today, isoScale is available at http://middle-down.github.io/Software/. More recently, we determined that the relative quantification of histone PTMs using middle-down MS proteomics is comparable to the more canonical bottom-up approach where shorter tryptic peptides are analyzed [27], demonstrating that middle-down MS is a reliable strategy for histone PTM quantification.

So far, middle-down MS has been exclusively performed using label-free approaches, as labeling adds further complexity into an already complex identification and quantification process. Metabolic “pulse” labeling was applied in a wide variety of histone-related projects using bottom-up MS, including determination of PTM catalysis rates on newly synthesized histones (using heavy lysines or arginines) [28], or the dynamics of histone methylations [29–31] [using heavy methionine, the precursor of the methyl donor *S*-adenosylmethionine (SAM)]. Combining middle-down MS and metabolic labeling would be the first technique that allows discrimination of newly synthesized proteins or PTMs while preserving the information of coexisting PTM patterns. Interestingly, a few studies demonstrated that analysis of intact proteins, namely top-down MS, can be performed when such proteins are metabolically labeled [32–34]. However, histone analysis is a whole different issue, due to their very large degree of modified forms, many of them leading to the same intact protein mass.

In this work, we labeled metabolically HeLa cells and cells undergoing epithelial to mesenchymal transition (EMT) using heavy lysine/arginine (K(^13^C_6_, ^15^N_2_), R(^13^C_6_, ^15^N_4_)) or methionine (M(methyl-^13^CD_3_)) in cell culture, respectively. Then, we analyzed histone H3 N-terminal tails using middle-down MS. We upgraded our in-house software isoScale [25] into the version isoScale labels (http://middle-down.github.io/Software/) to allow filtration of custom PTMs and heavy labeled peptide sequences, and then we demonstrate that middle-down MS can be used to analyze labeled histone N-terminal tails. Thanks to this method, we observed that H3K27me3 is a crucial player in EMT, and it is catalyzed during mesenchymal transition on histone proteins carrying the combinatorial pattern H3K27me2K36me2. This combination is converted into H3K27me3K36me2 in mesenchymal cells, likely silencing chromatin regions that were in a hybrid state in epithelial cells. In summary, our work enhances the flexibility of the middle-down MS platform, which can now be used to characterize the dynamics of both single and combinatorial histone PTMs.

## Methods

### Experimental design and statistical rationale

Equal amounts of HeLa cells grown in unlabeled or heavy KR (lysine + arginine) media were equally mixed prior middle-down MS analysis of histone H3 tails. Pearson’s correlation was assessed to determine biases in quantification due to heavy labeling. Confidence in EMT heavy methylation quantification was assessed by performing Pearson’s correlation of calculated relative abundances across technical and biological replicates, and between bottom-up and middle-down MS analysis (data reported in Additional file: Tables S1–3).

### HeLa S3 and EMT cells growth

HeLa cells were grown in suspension as previously described [35] and harvested using our standard protocol [36]. NMuMG cells (epithelial) were cultured in DMEM or DMEM in which normal lysine and arginine were replaced with the same amount of lysine(^13^C_6_, ^15^N_2_) and arginine(^13^C_6_, ^15^N_4_) and supplemented with 10% dialyzed FBS and penicillin–streptomycin antibiotics. To allow for the complete labeling of the histones with heavy K and R in both cell lines, we cultured the cells for six passages. Differentiation of heavy NMuMG cells was initiated with 5 ng/mL of TGFβ, while unlabeled NMuMG cells were treated with 5 ng/mL of DMSO. After two days of TGFβ treatment, equal amounts of non-differentiated and differentiated NMuMG cells were mixed. For the dynamic methylation study, NMuMG cells were cultured in DMEM medium supplemented with 10% dialyzed FBS and penicillin–streptomycin antibiotics at time zero. Then, normal methionine was replaced with the same amount of methionine-(methyl-^13^CD_3_) and cells were treated with 5 ng/mL of TGFβ. NMuMG cells were harvested at time zero, 24 and 48 h after TGFβ treatment.

### Histone purification from cells

Histone purification was performed as previously described [36]. Briefly, nuclei were isolated by suspending cells into nuclei isolation buffer (15 mM Tris–HCl (pH 7.5), 15 mM NaCl, 60 mM KCl, 5 mM MgCl_2_, 1 mM CaCl_2_, 250 mM sucrose, 0.2% NP-40) including the following inhibitors: 1 mM DTT, 0.5 mM AEBSF and 10 mM sodium butyrate. Nuclei were separated by centrifugation (1000g for 10 min), and 2 mL of cold 0.4 N H_2_SO_4_ was added on the nuclei pellet. Nuclei were incubated at 4 °C with shaking for 2 h. The nuclei were pelleted at 3400 g for 5 min, and proteins were precipitated from the supernatant with 25% TCA (w/v) for 1 h at 4 °C. The pellet was then washed with pure acetone to remove residual TCA.

### Histone H3 isolation and digestion

Purified total histones were resuspended in 0.1% trifluoroacetic acid (TFA) and loaded onto a 4.6 mm i.d. Vydac C_18_ column (218TP) using an off-line Beckman Coulter (System Gold) HPLC (Buffer A: 0.1% TFA, Buffer B: 95% acetonitrile, 0.08% TFA) at 0.8 mL/min as previously described [23]. HPLC–UV separation was performed using a gradient from 0 to 60% buffer B in 60 min, followed by from 60 to 100% buffer B in 10 min. Purified histone H3 was resuspended in 30 μL of 50 mM NH_4_HCO_3_, pH 8.0, and divided into two equal volumes, one for bottom-up and one for middle-down MS digestion. Bottom-up derivatization and digestion were performed as discussed in our freely available protocol [36] with minor modifications, i.e., NMuMG cells were digested only for 6 h instead of overnight. Trypsin was used at an enzyme/sample ratio of 1:20, overnight at room temperature. Middle-down MS samples were prepared by overnight digestion at room temperature with GluC at an enzyme/sample ratio of 1:20. The reaction was blocked by adding 1% formic acid for LC–MS analysis.

#### Bottom-up nano-LC-MS/MS and data analysis

SILAC-labeled EMT samples were quantified in two biological replicates. Heavy methyl-labeled EMT samples were analyzed in two technical replicates per time point. Samples were analyzed by using a nano-LC-MS/MS setup. The nano-LC was configured with a 75 µm ID × 17 cm ReproSil-Pur C_18_-AQ (3 µm; Dr. Maisch GmbH, Germany) nano-column using an EASY-nLC nano-HPLC (Thermo Fisher Scientific, San Jose, CA, USA). The HPLC gradient was 0–28% solvent B (*A* = 0.1% formic acid; *B* = 95% acetonitrile; 0.1% formic acid) over 40 min and from 28 to 80% solvent B in 5 min at a flow rate of 300 nL/min. The nano-LC was coupled with a Q-Exactive (Thermo Fisher Scientific, San Jose, CA, USA) mass spectrometer. Spray voltage was set at 2.3 kV and capillary temperature was set at 275 °C. Full-scan MS spectrum (*m*/*z* 290–1400) was performed in the Orbitrap with a resolution of 35,000 (at 200 *m*/*z*) with an AGC target of 10e6. The instrument operated in a data-independent acquisition (DIA) mode, as previously described in an Orbitrap Elite [37]. Fragmentation was performed with HCD normalized collision energy of 27, an AGC target of 5 × 10e5 and a resolution of 17,500. The intensity of isobaric peptides, i.e., peptides with the same mass but with PTMs on different positions on the amino acid sequence, was determined using fragment ions. Briefly, the intensity of the precursor ion signal was split between the isobaric forms according to the relative intensities of the fragment ion signals. Peak area was extracted from raw files by using our in-house software EpiProfile [38], which includes a pre-compiled list of peptides for quantification. The relative abundance of a given PTM was calculated by dividing its intensity by the sum of all modified and unmodified peptides sharing the same sequence.

#### Middle-down nano-LC-MS/MS and data analysis

SILAC-labeled HeLa samples used to assess quantification methods were analyzed in four technical replicates. SILAC-labeled EMT samples were analyzed in three technical replicates. EMT samples with heavy methylation labeling were analyzed in 1 or 2 replicates per condition. Samples were separated using an Eksigent 2D + nano-UHPLC (Eksigent, part of ABSciex). The nano-LC was equipped with a two-column setup, a 2-cm pre-column (100 µm ID) packed with C_18_ bulk material (ReproSil-Pur C18-AQ 3 µm; Dr. Maisch) and a 12-cm analytical column (75 μm ID) packed with Polycat A resin (PolyLC, Columbia, MD, 1.9 µm particles, 1000 Å). Loading buffer was 0.1% formic acid (Merck Millipore) in water. Buffer A and B were prepared according to Young et al. [23]. The gradient was delivered as follows: 5 min 100% buffer A, followed by 0–55% buffer B in 1 min, a nonlinear gradient from 55 to 85% buffer B in 120 min and 85–100% in 10 min. The flow rate for the analysis was set to 250 nL/min. MS acquisition was performed in an Orbitrap Fusion (Thermo) with a spray voltage of 2.3 kV and a capillary temperature of 275 °C. Data acquisition was performed in the Orbitrap for both precursor and product ions, with a mass resolution of 60,000 for MS and 30,000 for MS/MS. MS acquisition window was set at 660–720 *m*/*z*. Dynamic exclusion was disabled. Only charge state 8^+^ was accepted for MS/MS fragmentation. The isolation width was set at 2 *m*/*z*. The five most intense ions with MS signal higher than 5000 counts were isolated for fragmentation using ETD with an activation time of 20 ms. Three microscans were used for each MS/MS spectrum, and the AGC target was set to 2 × 10e5. Data processing was performed as described in Sidoli et al. [25]. Briefly, spectra were deconvoluted with Xtract (Thermo) and searched with Mascot (v2.5, Matrix Science, London, UK), including mono- and dimethylation (KR), trimethylation (K) and acetylation (K) as dynamic modifications. No fixed modifications were selected. The database used to search was human histones (UniProt release April 2014). Enzyme was GluC (cleaves after E) with 0 missed cleavages allowed. Mass tolerance was set to 2.1 Da for precursor mass and 0.01 Da for product mass; MS/MS tolerance set below the mass difference between acetylation and trimethylation (0.03 Da) allows score discrimination between the two PTMs and thus avoids wrong assignment of modifications (demonstrated in [25]). Heavy versions of the methylations were added to the Mascot server and to the search where needed; SILAC labeling was included for the metabolically labeled samples. Mascot result files were processed with isoScale labels [25] (http://middle-down.github.io/Software/) using a tolerance of 30 ppm, as we previously demonstrated it is a suitable value to filter confident identification and quantification [25]. Peptides with ambiguous modification site assignment were automatically discarded by the software. For quantification, the relative abundance of a given modified peptide (every combinatorial PTM pattern) was calculated by dividing the total ion intensity of this peptide by the sum of all modified and unmodified peptides sharing the same sequence. All raw files are available on https://chorusproject.org at the project no. 1287.

## Results

**Fig. 1:**
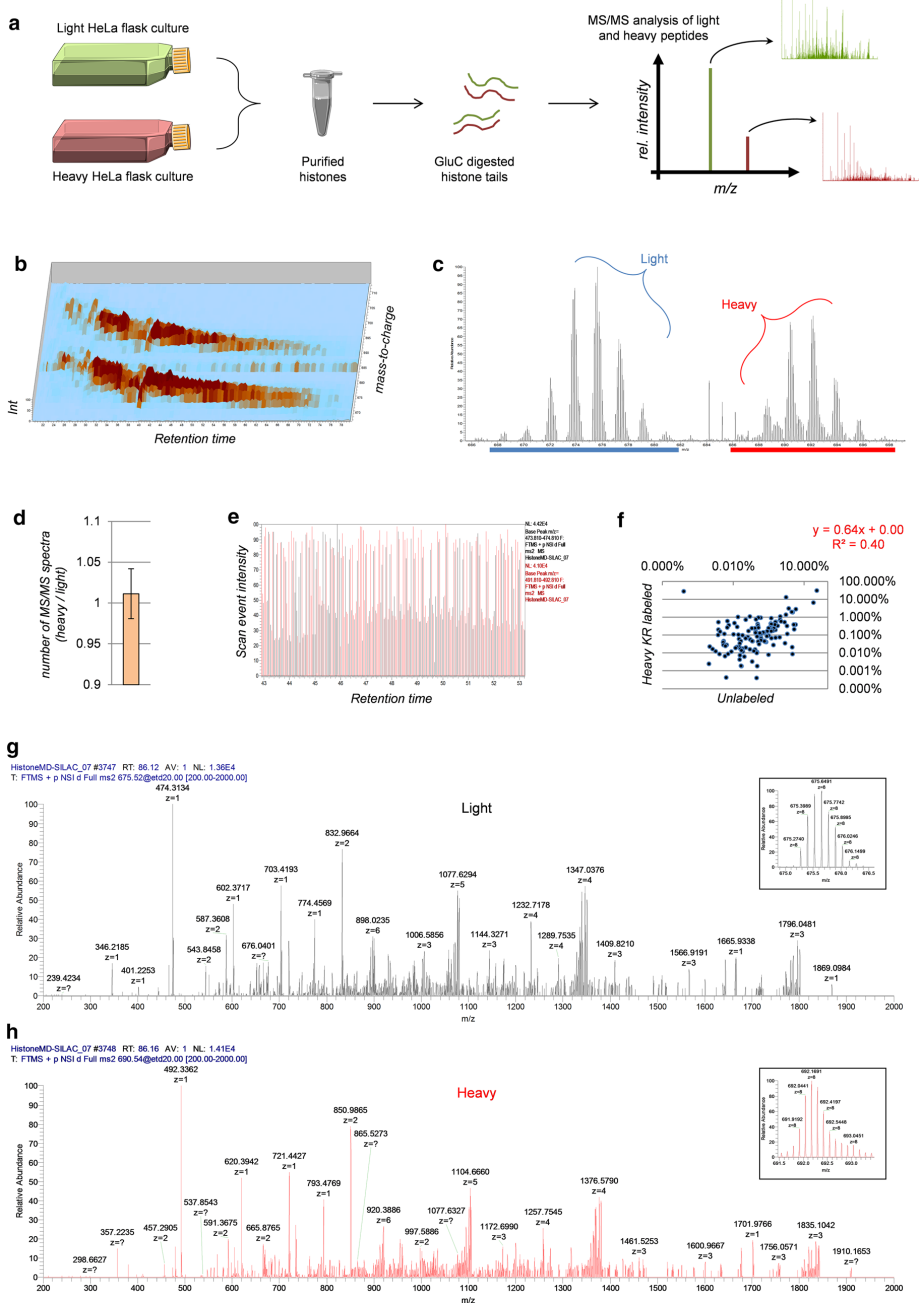
Analysis of histone H3 N-terminal tails from HeLa cells labeled with heavy lysine/arginine residues (heavy KR). **a** Workflow for metabolic labeling of histone sequences. Histones were extracted from HeLa S3 cells grown in equal amounts in light and heavy medium. After extraction, histone H3 was purified using C_18_ chromatography and digested using GluC. MS/MS-based quantification was performed using isoScale labels. **b** Nano-liquid chromatography (nLC)-MS ion map of the histone H3 N-terminal tail in its unlabeled and isotopically heavy labeled form. The *y* axis represents *m*/*z*, while the *x* axis represents retention time. The pattern above is the sequential elution of modified histone tails with heavy KR label, while the pattern below is the unlabeled tails. Histone tails elute from the most modified to the least modified, which is why the *m*/*z* value decreases as function of time. **c** Full MS spectrum representing differently methylated histone tails co-eluting from chromatography. Unlabeled (*light*) forms are *underlined in blue*, while heavy KR-labeled peptides are *underlined in red*. **d** Number of MS/MS spectra performed for light and heavy histone tails. Their ratio indicates that both species are equally subjected to MS/MS selection; *error bar* represents standard deviation of 4 replicates. **e** MS/MS events selecting either unlabeled (*black*) or heavy KR-labeled (*red*) peptides for fragmentation. No clustering of *colors* indicates that there is no bias in MS/MS selection for the two forms. **f** Correlation between quantified polypeptides for each of the two labels. Axes represent the average relative abundance of given modified polypeptides quantified in the two forms. Axes are Log_10_ scaled to facilitate visualization. **g** MS/MS ion spectrum of a selected histone tail in its unlabeled form and **h** the same histone tail in its heavy KR-labeled form. The MS scan of their precursor mass is displayed on the *square* at the *top right* of the MS/MS spectrum

**Fig. 2:**
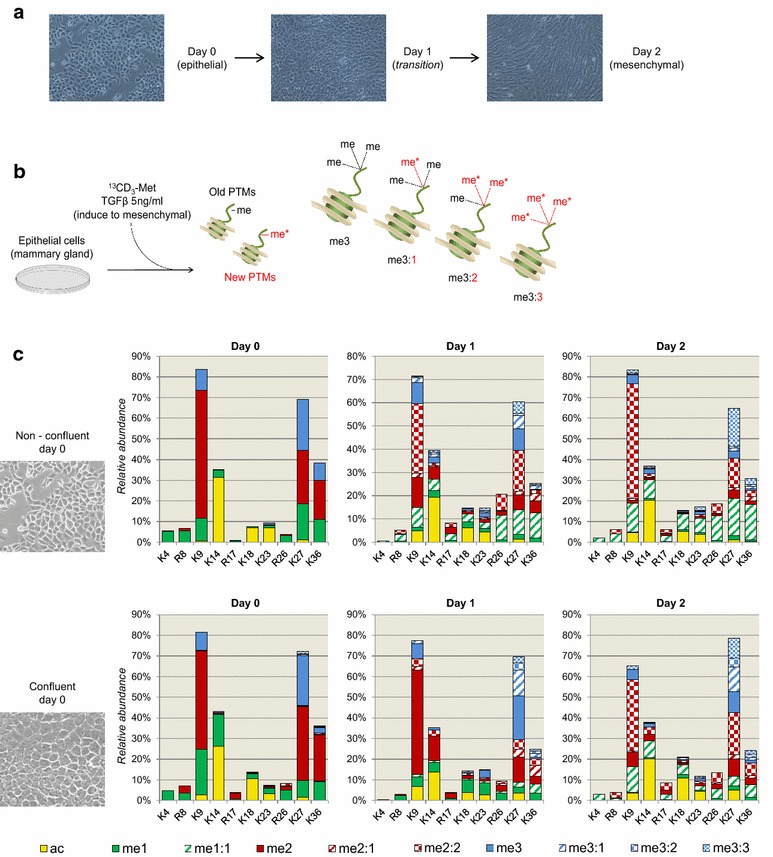
Heavy methyl labeling of epithelial to mesenchymal transition (EMT) cell lines. **a** Microscopy image of epithelial cell culture at days 0, 1 and 2. Day 0 is prior stimulation with TGFβ, inducing mesenchymal transition. **b** On the *left*, workflow displaying the treatment of epithelial cells to induce EMT. Briefly, epithelial cells were plated into a new media containing heavy labeled methionine. Newly synthesized PTMs are characterized by a mass shift of 4 Da, due to the replacement of CH_3_ with ^13^CD_3_. On the *right*, examples of the possible labeling combinations for trimethylation; the number of heavy labeled methyl groups is illustrated by the number after the colon (in *red*). **c** Relative abundance of single histone PTMs in EMT in not confluent culture (*top*) and confluent culture (*bottom*). Data are the average of three biological replicates (two for day 0). Heavy labeled methylations are highlighted by different degrees of *dashed bars*; one heavy methyl is indicated with * diagonal lines*, two heavy methyl groups with a cheeseboard-like theme and three heavy methyl groups with more dense tiny squares. The relative abundance of single PTMs was obtained by summing the relative abundance of all combinatorial forms containing each given PTM

In this work, we evaluated the feasibility of middle-down MS proteomics to characterize the dynamics of histone PTMs upon metabolic labeling. Stable isotope labeling in cell culture (SILAC) is a routine quantitative strategy in proteomics [39]; by differentially labeling the protein amino acid sequence it is possible to discriminate samples mixed in the early stage of the preparation. Stable isotope labeling of the PTMs has also been applied, but it is less widely adopted (e.g., [29–31]). To assess the reliability of middle-down MS in analyzing isotopically labeled histones, we first tested the capability of the technique in discriminating unlabeled versus heavy labeled peptide sequences. To do so, we mixed histone H3 from HeLa cells grown in unlabeled and heavy KR medium in 1:1 ratio, digested them using GluC and analyzed them using nLC-MS/MS with ETD fragmentation (Fig. 1a). After this analysis, we used isotopically labeled methionine to identify newly synthesized methylations on histone tails, being methionine a precursor of methylation. To do so, we monitored the accumulation of heavy labeled methylation on epithelial cells transitioning into mesenchymal cells at days 0, 1 and 2 (Fig. 2b). Overall, this study allowed us to evaluate the quantification accuracy of middle-down MS in the analysis of metabolically labeled histone samples.

### Reproducibility of PTM quantification in unlabeled and heavy labeled histone H3 tails

First, we aimed to show that metabolic labeling of the histone sequence does not generate a significant bias in the analysis of intact N-terminal tails. We mixed 1:1 unlabeled and heavy (KR) labeled histone H3, digested them using GluC and run them with a rather short (90 min) gradient compared to conventional middle-down MS protocols [23, 25]. The nLC ion map showed that both unlabeled and heavy KR-labeled species were separated with very comparable efficiency (Fig. 1b), as they generated two distinct traces with highly comparable trends. The MS signals were also highly distinct; since histone H3 N-terminal tails include 10 lysine residues and 6 arginine residues, a fully heavy KR-labeled histone tail has a mass difference of about 134 Da (16.75 *m*/*z* for charge state 8^+^) from the unlabeled form (Fig. 1c). Differently methylated forms are not fully resolved using WCX–HILIC chromatography [25], reducing the gap between labeled and unlabeled species. However, the overwhelming mass distance between unlabeled and heavy KR-labeled tails guarantees MS signals not to overlap nevertheless.

As compared to short peptides typical of bottom-up MS, heavy labeled histone tails are hardly 100% labeled, which is a common issue for long protein sequences as illustrated by Collier et al. for top-down MS [33]. Because of this, the isotopic distribution of heavy labeled sequences is broader than unlabeled forms, reducing the relative intensity of any given isotope (Fig. 1c, g, h). This widening of the isotopic distribution could create a bias in favor of unlabeled histone tails for selecting them for fragmentation, in the quality of fragmentation, and therefore identification. Our data demonstrate that no bias is present in MS/MS selection; the number of MS/MS in each run was equivalent for the unlabeled and the heavy forms (Fig. 1d, calculated based on peptide spectral counts). Moreover, those scans alternated selection of heavy versus light peptides in a rather constant manner (Fig. 1e), highlighting that there was no clustering of scans for the same labeling type within a given time range. In summary, heavy KR-labeled forms were chosen for fragmentation as frequently as unlabeled forms, demonstrating that the widening of the isotopic distribution did not impart a bias for peptide selection.

MS/MS spectra of histone H3 N-terminal tails were identified with Mascot and filtered/quantified using our free version of isoScale labels (http://middle-down.github.io/Software/). Results showed a sufficiently linear correlation of abundances (*R* ^2^ = 0.4; Fig. 1f), which is what we expected after mixing in a 1:1 light/heavy ratio identical cultures of HeLa cells. Although this might seem not an impressive achievement, it is important to underline that every data point in Fig. 1f plot (*N* = 131) is a different combinatorial PTM form quantified in both its unlabeled and heavy KR-labeled state; quantification of labeled hypermodified polypeptides is currently a prohibitive analysis for any other technique. Quantification of middle-down MS data was performed by summing the MS/MS ion intensities specific to a certain modified form, as described in [25]. Together, these data demonstrate that isotopically labeled amino acid residues do not drastically affect MS selection, peptide identification and quantification.

### Turnover of histone H3 during epithelial to mesenchymal transition

We then applied metabolic labeling to cells undergoing epithelial to mesenchymal transition (EMT). First, we verified absence of bias in the labeling by mixing epithelial cells grown for six passages in light medium versus six passages in heavy KR medium in a 1:1 ratio and analyzed them using the more traditional bottom-up approach [40]. Results from two biological and three technical replicates showed an average heavy/light ratio of 1.42 (Additional file: Table S1), tuned down to the more ideal 1.099 heavy/light ratio after removing the outlier H3.3K27me2K36me2. This result, although significantly higher than the expected ratio 1 (two-tail heteroscedastic *t* test, *p* value <0.05), confirmed that heavy labeling incorporation was efficient and nearly complete. High reproducibility between replicates was assessed using Pearson’s correlation (avg correlation = 0.86; Additional file: Table S1), and the significance of the correlation was assessed using the *t* test (*p* value of the avg correlation = 0.027). Peptides were quantified using our in-house software EpiProfile [38]. After assessing efficient labeling, we used the EMT model to analyze and compare old (unlabeled) versus newly synthesized (heavy KR labeled) histones during mesenchymal transition. To do so, we transferred epithelial cells previously grown in normal unlabeled media into a heavy KR media while inducing transition with TGFβ (5 ng/mL) (Additional file: Fig. S1a). Microscopy analysis confirmed that after two days the cells acquired the mesenchymal phenotype (Fig. 2a). We purified histone H3 using HPLC–UV C_18_ chromatography, digested it using GluC and analyzed it using our middle-down MS workflow. Results confirmed that middle-down MS provided the sensitivity to characterize 183 different combinations of PTMs in unlabeled and heavy KR-labeled histone H3 tails (Additional file: Table S2). Pearson’s correlation between replicates (Additional files: Fig. S1b and Table S2) was on average 0.81 for newly synthesized (heavy) histone tails and 0.52 for old (light) histone tails; this last did not pass the correlation significance threshold. By using the relative abundances of the identified combinatorial PTMs, we deconvoluted the relative abundance of the individual histone marks, i.e., the abundance of a given single PTM is obtained by summing all the relative abundances of polypeptides carrying that PTM. The relative abundance of single PTMs was highly comparable when comparing, e.g., days 1 and 2 on newly synthesized histones (Additional file: Fig. S1c). All bar graphs represent the relative abundance of the PTMs; the remaining percentage (adding up to 100%) is the calculated unmodified state.

Next, we aimed to assess the ability of middle-down MS in identifying and quantifying heavy labeled methylations. To do so, we set up an experiment with EMT in an analogous manner as just described. Briefly, we plated epithelial cells grown in unlabeled media into a media containing heavy labeled methionine and the factors that induce mesenchymal transition (Fig. 2b). Methionine is a precursor of *S*-adenosyl methionine (SAM), which is the source of methyl groups for methyltransferases that catalyze methylation on lysine and arginine residues. Analysis of methylation is further complicated by the fact that methylation can occur in multiple degrees from monomethylation up to trimethylation. For convention, we indicated the number of heavily labeled methyl groups by a number after colon (Fig. 2b); for example, a trimethyl mark with two heavy labeled methyl groups has the code me3:2. In principle, the bioinformatics pipeline we have established should minimize errors in the discrimination of canonical versus heavy labeled methylation; both the MS/MS ion tolerance during database searching (set to 0.01 Da) and the filtering using isoScale (discards every identified peptide without unique fragment ions for unambiguous localization of the PTM with a tolerance of 30 ppm) proved effective in efficiently discriminating trimethylation (42.047 Da) from acetylation (42.011 Da) [25]. The mass difference between unlabeled and labeled methylation is about 4 Da, which is far larger than me3/ac. Nevertheless, we manually inspected examples of identifications of peptides carrying heavy labeled methylations to ensure characterization confidence (Additional file: Figs. S2–5). In selected examples, the average MS/MS fragment ion mass deviations were all within 3 ppm, with no remarkable differences if they were canonical methylations (e.g., K27me2; Additional file: Fig. S2), or heavy labeled methylations (e.g., K27me2:2; Additional file: Fig S3), or multiply modified peptides (e.g., K14acK27me2:2 and K23me1:1K27me2:2; Additional file: Figs. S4 and S5, respectively). Filtering polypeptides with such modifications does not necessarily require isoScale labels, but it can be performed with the already published isoScale slim [41]; isoScale slim treats the heavy labeled methyl forms like any other modification, implying that no restriction to canonical PTMs is applied when using the software.

Histone H3 N-terminal tails were quantified at three time points of non-confluent EMT cells: before plating epithelial cells in heavy methionine medium, after 1 day and after 2 days of mesenchymal transition (Additional file: Table S3). Heavy labeled methylation showed increased incorporation at days 1 and 2, as expected (Fig. 2c). Every histone H3 PTM site investigated (K4, R8, K9, K14, K18, K23, R26, K27, K36) showed an overall downward trend of canonical methylation and upward trend of their respective heavy labeled forms (Additional file: Fig. S6). As validation, we repeated the experiment using confluent EMT cells, when growth is reduced due to contact inhibition and thus PTM turnover. Results showed that faster duplicating cells (non-confluent) incorporated heavy labeled methylation more rapidly than slow-growing cells (Fig. 2c top and bottom, respectively). Specifically, at day 1 the total percentage of peptides containing at least one heavy labeled methyl group was 81.5 in non-confluent cells and 56.1 in confluent cells (Additional files: Fig. S7 and Table S3); at day 2, the percentages were 96.3 and 87.3, respectively. H3K27me3 was the most rapid trimethyl mark detected as heavy labeled by observing the two experiments combined. This was remarkably different from, e.g., the other major repressive mark H3K9me3; after two days of mesenchymal transition, the majority of H3K9me3 was still not isotopically labeled in both experiments (Fig. 2c top and bottom).

**Fig. 3:**
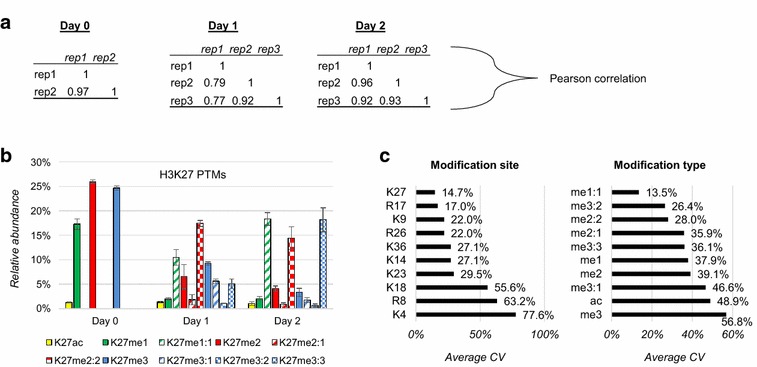
Reproducibility of PTM quantification (including heavy labeled methylations) across the biological replicates of the non-confluent EMT cell experiment. **a** Pearson’s correlation of biological replicates for each of the three time points. **b** Relative abundance and standard deviation (represented as *error bars*) of the modified forms of H3K27. Figure illustrates the variation of the biological replicates. *Color* and theme coding is the same as Fig. 2c. **c** Coefficient of variation (CV) of the biological replicates grouped by modification site and modification type. The average across the three time points was taken

To assess reproducibility of the analysis, we performed Pearson’s correlation across the biological replicates (Fig. 3a). No technical replicates were performed in this experiment. Results showed significant correlation (*t* test *p* value < 0.05) and low variability of the analysis. As example, we highlighted the relative abundance and the standard deviation across biological replicates of the modified forms of H3K27 (Fig. 3b). The other marks are all illustrated in Additional file: Fig. S6. We also represented the overall coefficient of variation (CV) for all single marks of histone H3 investigated in this study, grouped by variability of the modification site and modification type (Fig. 3c). Together, these results demonstrate that middle-down MS coupled to metabolic labeling can quantify differences in PTM catalysis rates, and potentially unravel dynamic biochemical properties of chromatin. Next, we verified that the results we obtained are comparable with results obtained by performing bottom-up MS on the same samples.

### Comparison with bottom-up MS results

**Fig. 4:**
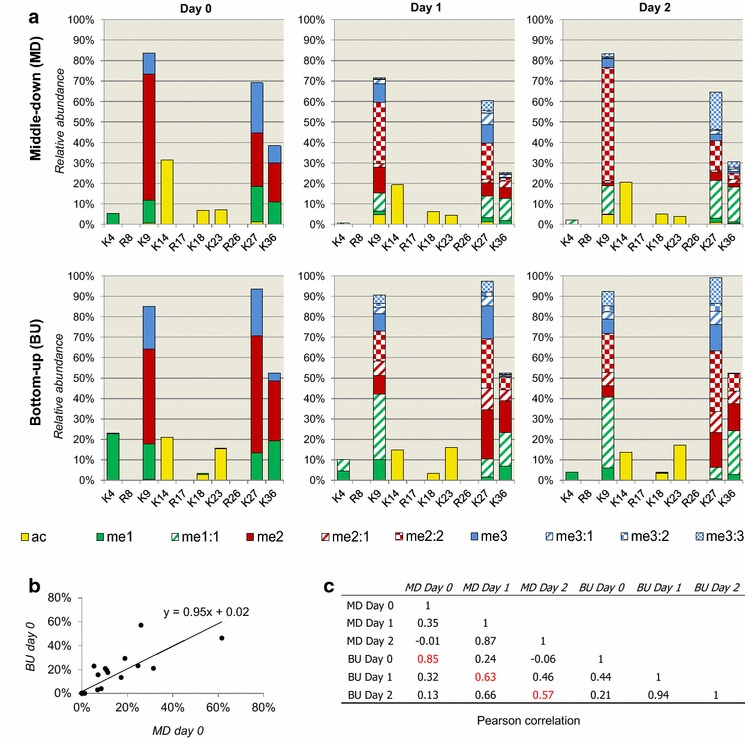
Comparison of single PTM quantification obtained from the middle-down and the bottom-up MS analysis. **a** Relative abundance of single histone PTMs quantifiable by both middle-down (*top*) and bottom-up (*bottom*) MS strategies. The relative abundance of single PTMs was obtained by summing the relative abundance of all combinatorial forms containing each given PTM. **b** Example of correlation between the day 0 results of bottom-up and middle-down MS. **c** Pearson’s correlation values between the described runs. Averages of biological replicates were used for the comparison; in *red*, correlation of the same time point of middle-down versus bottom-up MS

Quantification of middle-down-sized peptides is computationally challenging; multiple isobaric forms are not chromatographically separated and thus lead to mixed MS/MS spectra. Assessing the quantification confidence of middle-down MS is not a trivial task, as testing the method on a library of synthetic polypeptides of 50 amino acid residues would be prohibitively expensive. Because of this, we decided to assess the performance of the method by analyzing the same sample using the more traditional bottom-up MS strategy [36]. Bottom-up MS is currently the golden standard in histone PTM analysis and, more generally, in proteomics. However, the comparison with middle-down MS results should consider a number of limitations: (1) Only the relative abundance of single, and not combinatorial, PTMs can be assessed; (2) accurate relative abundance of arginine methylation cannot be estimated by bottom-up MS, being the cleavage site of the enzyme during sample preparation; (3) peptide ionization efficiency is more pronounced in bottom-up MS (as discussed in [27]) and thus extremely similar results are unlikely nevertheless; (4) the software we currently employ for bottom-up MS analysis, EpiProfile [38], is currently not trained to quantify all methylated forms on the sites K14/K18/K23, due to the large variety of isobaric peptides that are potentially present. Considering only the PTMs detectable by both techniques, we analyzed non-confluent EMT cells at the three time points and compared the two sets of results by averaging the biological replicates. Results showed a good reproducibility between bottom-up and middle-down MS (Fig. 4a and Additional file: Table S3). Specifically, at day 0 we observed the most similar results in terms of both slope (Fig. 4b) and correlation (Fig. 4c). At days 1 and 2, we obtained an acceptable, although not significant, correlation. This was not surprising, as the PTM complexity in days 1 and 2 is far higher than in day 0, and thus accurate quantification is more challenging. Interestingly, the correlations obtained for each of the time point investigated were all more accurate than our previous comparison between bottom-up and middle-down MS (*R* ^2^: 0.47) [27], where we showed that both methods achieved comparable accuracy in determining PTM relative abundance and stoichiometry. This suggests that the incremental optimization applied to the middle-down MS strategy is moving the technique toward higher quantification accuracy.

### Analysis of H3K27me3 dynamics and coexistence frequencies during EMT

**Fig. 5:**
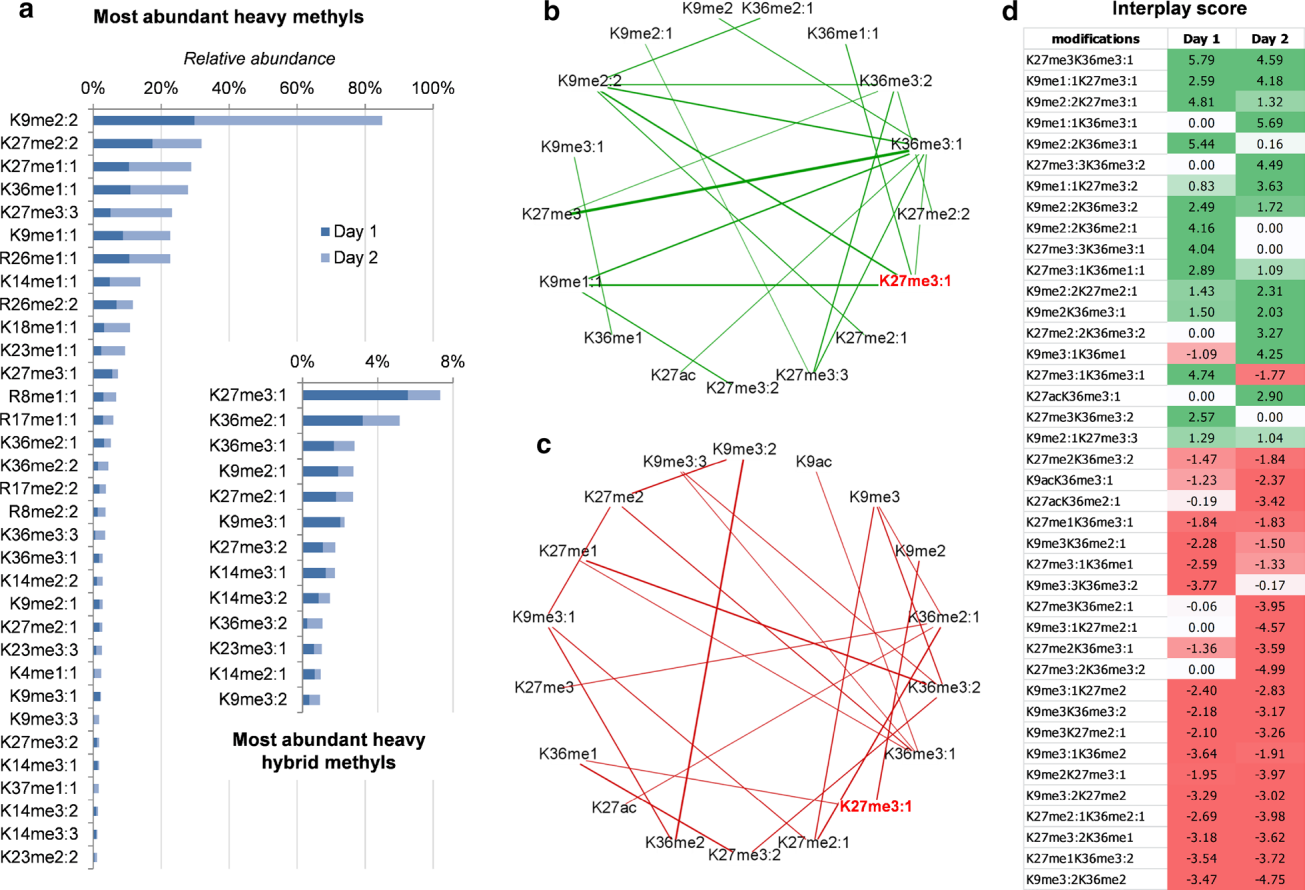
Analysis of heavily labeled methylations at days 1 and 2 of EMT. **a** Relative abundance of heavy labeled methylations, sorted by cumulative intensity for the days 1 and 2. In the smaller bar plot, zoom for the hybrid methylations, i.e., methylated states containing both unlabeled and heavy labeled methyl groups.*Ring graph* representing **b** positive and **c** negative interplay scores between methylations on K9, K27 and K36 sites. *Line thickness* represents interplay absolute values. High interplay values indicate that the * two marks* coexist on the same histone tail with a frequency higher (if positive) or lower (if negative) than random co-occurrence. **d** Most intense absolute interplay scores that include at least one *hybrid mark*

Finally, we aimed to show a potential approach to analyzing such complex dataset and provide biological insights for the EMT cell model. To do so, we focused on the analysis of the “hybrid” methylations, which are PTMs only partially heavily labeled, i.e., me2:1, me3:1 and me3:2. In this specific data analysis, we did not consider fully labeled forms, as they cannot be easily discriminated from simple PTM turnover, i.e., not regulatory events. Hybrid methylations benchmark a PTM site that inherited part of the methylation state from day 0 (unlabeled) and part from the heavy labeled methionine, only present after induction into mesenchymal transition. For instance, a trimethylation type me3:1 contains two light methyl groups (with every probability from day 0) and one heavy methyl group (acquired during the transition) (Fig. 2b). From this analysis, we observed that H3K27me3:1 was the most abundant hybrid PTM (Fig. 5a). Other abundant hybrid marks were K36me2:1 and K36me3:1. This showed that during mesenchymal transition some regions of the chromatin did not simply replace old H3K27me3 with new H3K27me3, but other regions modified H3K27me2 into H3K27me3.

To define in which regions H3K27me2 was converted into H3K27me3, we analyzed the most frequent coexistences of H3K27me3:1 in day 1 and day 2 with other PTMs. To do so, we calculated the relative abundance of all binary combinations of histone PTMs by summing the relative abundance of all peptides carrying the two PTMs. This relative abundance was then converted into an “interplay score” as previously described [42]. Briefly, the observed coexistence frequency was divided by a predicted coexistence frequency, calculated by multiplying the relative abundance of the two single PTMs by each other. This normalization provides a score that is positive if the two PTMs coexist on the same histone tail for a frequency higher than just random chance and negative if they are mutually exclusive from the same histone. The most positive interplays of H3K27me3:1 were H3K9me1:1, H3K9me2:2, H3K36me1:1 and H3K36me3:1 (Fig. 5b). This implies that H3K27me2 is most frequently converted into H3K27me3 on histone tails where H3K27me2 is mostly flanked by H3K36me2 and no modifications on H3K9. Interestingly, H3K36me2 is known to frequently occur in gene bodies of actively transcribed genes, meaning that the conversion of H3K27me2 into H3K27me3 during EMT could be a mechanism of gene silencing for the chromatin domains including these histones. The most negative interplays of H3K27me3:1 were H3K36me1 and H3K9me2 (Fig. 5c), confirming that the transition of H3K27me2 into H3K27me3 does not occur on histones already modified with H3K9 methylation. The most pronounced interplays including hybrid methylation marks is displayed in Fig. 5d; results in day 1 and day 2 show high consistency, implying that interplays are cell stage independent, as we previously confirmed [41, 42]. In these previous studies, we also confirmed in mammal embryonic stem cells that PTMs such as H3K27me3K36me3 and H3K9me1K27me3 rarely coexist on the same histone tail. Interestingly, in the current study we identified these two as the top positive interplays for hybrid methylations (Fig. 5d), i.e., H3K27me3K36me3:1 and H3K9me1:1K27me3:1. Their predicted modification state at day 0, i.e., without the heavy labeled methyls, would be H3K27me3K36me2 and H3K9unmodK27me2; these combinations are very frequent in the previously investigated cell lines [41, 42], suggesting that the combinatorial PTM patterns in mesenchymal cells are aberrant and atypical of “healthy” cell lines. More studies should be performed, but overall this suggests another possible approach to the analysis of such dataset. Collectively, we demonstrated that metabolic labeling can be combined with middle-down MS to observe regulatory events of combinatorial PTMs at an unprecedented depth of information.

## Discussion

In this manuscript, we demonstrate that middle-down MS proteomics can be exploited to identify and quantify combinatorial histone PTMs in the presence of either stable isotopic labeling of the protein sequence or the PTM methylation. Such stable isotopic labeling was introduced in the cells by using amino acids for the cell metabolism; for labeling the protein sequence, we used heavy lysine and arginine (typical of SILAC proteomics), while for labeling the methyl groups we used heavy methionine. First, we assessed that hypermodified histone tails can be equally identified and quantified in the presence of with these isotopic labels. Next, we verified that middle-down MS results are relatively comparable to bottom-up results, albeit with some variability. Finally, we exploited this new technology to define the dynamics of H3K27me3 during epithelial to mesenchymal transition, highlighting that this modification is mostly catalyzed on histones carrying specific PTM patterns.

Bottom-up MS is currently the most adopted method for histone and histone PTM characterization. As such, bottom-up MS methods and analysis tools are widely available. However, in bottom-up MS the connectivity between most PTMs is lost upon trypsin digestion. Middle-down MS offers an attractive alternative because we can analyze the intact tail. This methodology enables the identification of nearly all combinatorial PTM profiles, as most PTMs reside on the histone N-terminal tail. However, middle-down MS is still a more challenging and less reproducible technique, mostly due to the different type of chromatography, high performance MS and bioinformatics required. Such imperfect chromatography leads to a large percentage of isobaric combinatorial PTMs present in mixed MS/MS spectra, which are hard to identify and differentially quantify.

Because of the existing challenges, middle-down MS analyses have all been performed using label-free quantification so far. Very recently, Liao et al. [43] showed that by performing propionic anhydride derivatization on middle-down-sized histone tails you can improve separation with reversed-phase chromatography. It is not yet proved that derivatization with differentially labeled propionic anhydride can be performed to multiplex samples, but it is a possibility on the horizon. Sample labeling is an appealing goal, as label-free techniques require the most instrument time due to the impossibility of multiplexing, and they are more susceptible to variations in sample preparation and instrument performance. But mostly, metabolic labeling has one unique advantage, which is the possibility to “pulse” stable heavy isotopes and monitor the dynamics of biological processes such as protein and PTM turnover. We exploited this aspect in the present work by proposing a new analysis of histone PTMs with unprecedented depth. In fact, our workflow proved to be able to characterize PTM turnover and predict PTM cross talk. By using EMT as model system, we identified the binary marks H3K27me2H3K36me2 and H3K9unmodK27me2 being the main precursors of the transition into the chromatin silenced state H3K27me3K36me2 and H3K9me2K27me3 during EMT. This would have not been possible with either label-free middle-down MS or the traditional bottom-up MS, even if stable isotope labeling was adopted. We speculate that this type of analysis has potential in answering questions such as the order of deposition or removal of PTMs on newly synthesized or old histones, which will be critical for elucidation of PTM propagation and inheritance. Studying combinatorial PTM turnover during different cellular processes such as differentiation will also enable us to determine how chromatin states change. For example, a hypothetical combination such as H3K14acK18acK27me3:2 would indicate that the chromatin was in an open state (acetylation is correlated with euchromatin), but introduction of K27me, a repressive mark, could poise the chromatin to a more closed state. In the present manuscript, we provide the full workflow for nLC-MS/MS analysis and provide the link to our raw data and freely available software isoScale labels (http://middle-down.github.io/Software/) for filtering and quantification of middle-down MS identifications.

This methodology is also widely applicable to the other histone variants, or proteins other than histones. It will undoubtedly also be useful in quantifying turnover of protein variants that have a small number of amino acid substitutions such as histone H3.1 versus H3.3, as by using middle-down MS we can define the sequence variation of these two highly similar variants on the histone tail, not possible with most peptides in bottom-up MS. However, it is important to keep into consideration that when analyzing peptide sequences containing methionine residues the labeling of the protein sequence must be taken into account when analyzing methylation turnover, due to the fact that labeling of methyl groups is performed by using isotopically heavy methionine. Finally, the combination of protein and PTM labeling within a single experiment could provide a means to monitor new PTM deposition on old versus new protein, although this type of double labeling would require further validation of results due to the larger variety of the molecular candidates potentially present in the sample. This type of analysis may require a more advanced version of isoScale, or other software for middle-down MS. There are many potential applications of middle-down MS coupled to metabolic labeling, and this method will be particularly useful for highly modified proteins where maintaining PTM connectivity is desirable.

## Conclusions

Collectively, we demonstrate that histone H3 N-terminal tails can be analyzed using middle-down MS when metabolic labeling is performed. In our work, we applied the technique for the investigation of histone PTM patterns during EMT, showing that H3K27me3 in mesenchymal cells is mostly the product of the catalysis on histones modified in specific ways in epithelial cells. We provide the workflow and the software to perform such experiment and filter result files. We consider this method of high potential to explore the dynamics of histone PTMs from a different perspective, not currently possible with any other technique. Our personal opinion is that middle-down MS should be more promoted as tool for chromatin biology, since it has great potential but it is still scarcely exploited. In this manuscript, we show that the methodology has reached an additional level of flexibility, potentially attractive for the above-mentioned applications.

## Additional files

**Additional file 1: Figure S1.** Analysis of EMT cells labeled using heavy lysine/arginine (heavy KR) during mesenchymal transition. (A) Workflow for metabolic labeling of EMT cells at the histone sequence level (heavy KR). (B) Pearson’s correlation values between technical and biological replicates (rep 2-1 implies second biological, first technical), differentiated between combinatorial PTMs quantified on unlabeled histone tails (old histones) and heavy labeled histone tails (new). (C) Bar plot representing the relative abundance of single histone PTMs on heavy labeled histone tails at day 1 (top) and day 2 (bottom). Unlabeled histone tails were excessively low abundant to provide a confident quantification of single histone PTMs. **Figure S2.** Annotated MS/MS spectrum of the histone H3 N-terminal tail modified as K27me2. From the top to bottom, (i) annotated sequence with highlights of identified fragments and modified site, (ii) annotated spectrum, (iii) observed mass, identified modification and Mascot (Matrix Science) identification scores, (iv) distribution of the fragment mass error, expressed in Da (left) and ppm (right). **Figure S3.** Annotated MS/MS spectrum of the histone H3 N-terminal tail modified as K27me2:2. From the top to bottom, (i) annotated sequence with highlights of identified fragments and modified site, (ii) annotated spectrum, (iii) observed mass, identified modification and Mascot (Matrix Science) identification scores, (iv) distribution of the fragment mass error, expressed in Da (left) and ppm (right). **Figure S4.** Annotated MS/MS spectrum of the histone H3 N-terminal tail modified as K14acK27me2:2. From the top to bottom, (i) annotated sequence with highlights of identified fragments and modified site, (ii) annotated spectrum, (iii) observed mass, identified modification and Mascot (Matrix Science) identification scores, (iv) distribution of the fragment mass error, expressed in Da (left) and ppm (right). **Figure S5.** Annotated MS/MS spectrum of the histone H3 N-terminal tail modified as K23me1:1K27me2:2. From the top to bottom, (i) annotated sequence with highlights of identified fragments and modified site, (ii) annotated spectrum, (iii) observed mass, identified modification and Mascot (Matrix Science) identification scores, (iv) distribution of the fragment mass error, expressed in Da (left) and ppm (right). **Figure S6.** Relative abundance of all quantified methylations during EMT stimulation combined with heavy methyl labeling. Each plot represents the trend line of differently methylated PTM sites. Error bar represents standard deviation between three biological replicates (two for day 0). **Figure S7.** Sum of all quantitative values of peptides identified in the EMT cell lines incubated with heavy methionine during transition. The relative abundances represent the total quantification of peptides carrying no heavy labeled methylations (in blue) versus peptides carrying at least one heavy labeled methylation (in orange). Evidently, cells growing in a non-confluent state acquire heavy methylation faster than confluent cells, which have reduced growth rate.

**Additional file 2: Table S1.** Bottom-up MS quantification of epithelial cells grown in light and heavy KR medium and mixed 1:1. Relative abundance of bottom-up-sized histone peptides labeled with light and heavy KR. The first columns display the relative abundance of the same peptide in unlabeled (light) versus heavy KR-labeled form. The columns H/L ratio represents the ratio of the peptides between heavy and light. On the top right, average of H/L ratios across all detected peptides and respective standard deviation. On the bottom right, correlation analysis and significance estimated using the *t* test.

**Additional file 3: Table S2.** Quantification of middle-down-sized polypeptides during EMT. Labeling is illustrated in Additional file: Fig. S1a; briefly, epithelial cells were plated into heavy KR media during mesenchymal transition. Unlabeled and heavy KR-labeled histone tails are defined as old and new, respectively. Two biological replicates were performed for each experiment. On the right, correlation analysis and significance estimated using the *t* test.

**Additional file 4: Table S3.** Relative abundance of combinatorial PTMs in the EMT experiment including heavy methylation labeling. Labeling is illustrated in Fig. 2b; briefly, epithelial cells were plated into heavy methionine media during mesenchymal transition. Biological replicates were performed for the not confluent experiment. On the right, deconvoluted abundance of single histone marks. On the further right, alignment with results obtained from the bottom-up analysis on the same samples. Confluent experiment results are listed in the second sheet.

## Abbreviations

EMT: epithelial to mesenchymal transition
ETD: electron transfer dissociation
KR: lysine/arginine (used when mentioned stable isotopic labeling)
MS: mass spectrometry
MS/MS: tandem mass spectrometry
nLC: nano-liquid chromatography
PTM: posttranslational modification
SAM: *S*-adenosylmethionine
WCX–HILIC: weak cation exchange–hydrophilic interaction chromatography
