# Supplementary material for: Metabolic labeling in middle-down proteomics allows for investigation of the dynamics of the histone code
Source: Epigenetics Chromatin. 2017 Jul 6;10:34. doi: 10.1186/s13072-017-0139-z (PMC5501349; doi:10.1186/s13072-017-0139-z)
Supplement: Supplementary file 1 — Additional file 1: Figure S1. Analysis of EMT cells labeled using heavy lysine/arginine (heavy KR) during mesenchymal transition. (A) Workflow for metabolic labeling of EMT cells at the histone sequence level (heavy KR). (B) Pearson’s correlation values between technical and biological replicates (rep 2-1 implies second biological, first technical), differentiated between combinatorial PTMs quantified on unlabeled histone tails (old histones) and heavy labeled histone tails (new). (C) Bar plot representing the relative abundance of single histone PTMs on heavy labeled histone tails at day 1 (top) and day 2 (bottom). Unlabeled histone tails were excessively low abundant to provide a confident quantification of single histone PTMs. Figure S2. Annotated MS/MS spectrum of the histone H3 N-terminal tail modified as K27me2. From the top to bottom, (i) annotated sequence with highlights of identified fragments and modified site, (ii) annotated spectrum, (iii) observed mass, identified modification and Mascot (Matrix Science) identification scores, (iv) distribution of the fragment mass error, expressed in Da (left) and ppm (right). Figure S3. Annotated MS/MS spectrum of the histone H3 N-terminal tail modified as K27me2:2. From the top to bottom, (i) annotated sequence with highlights of identified fragments and modified site, (ii) annotated spectrum, (iii) observed mass, identified modification and Mascot (Matrix Science) identification scores, (iv) distribution of the fragment mass error, expressed in Da (left) and ppm (right). Figure S4. Annotated MS/MS spectrum of the histone H3 N-terminal tail modified as K14acK27me2:2. From the top to bottom, (i) annotated sequence with highlights of identified fragments and modified site, (ii) annotated spectrum, (iii) observed mass, identified modification and Mascot (Matrix Science) identification scores, (iv) distribution of the fragment mass error, expressed in Da (left) and ppm (right). Figure S5. Annotated MS/MS spectrum of the [file 13072_2017_139_MOESM1_ESM.pdf]

Supporting information

Metabolic labeling in middle-down proteomics allows for investigation of the dynamics of the histone code

Simone Sidoli[1], Congcong Lu[1], Mariel Coradin[1], Xiaoshi Wang[1], Kelly R. Karch[1], Chrystian Ruminowicz[2] and Benjamin A. Garcia[1]*

[1]Epigenetics Institute, Department of Biochemistry and Biophysics, Perelman School of Medicine, University of Pennsylvania, Philadelphia, PA 19104, USA

[2]Private developer, Białystok, Poland

*Corresponding author:

Benjamin A. Garcia, Epigenetics Institute, Department of Biochemistry and Biophysics, Perelman School of Medicine, University of Pennsylvania, Room 9-124, 3400 Civic Center Blvd, Bldg 421, Philadelphia, PA 19104, USA. E-mail: bgarci@mail.med.upenn.edu; Phone: 1-215-573-9423; Fax: 215-573-4764

Figure S1

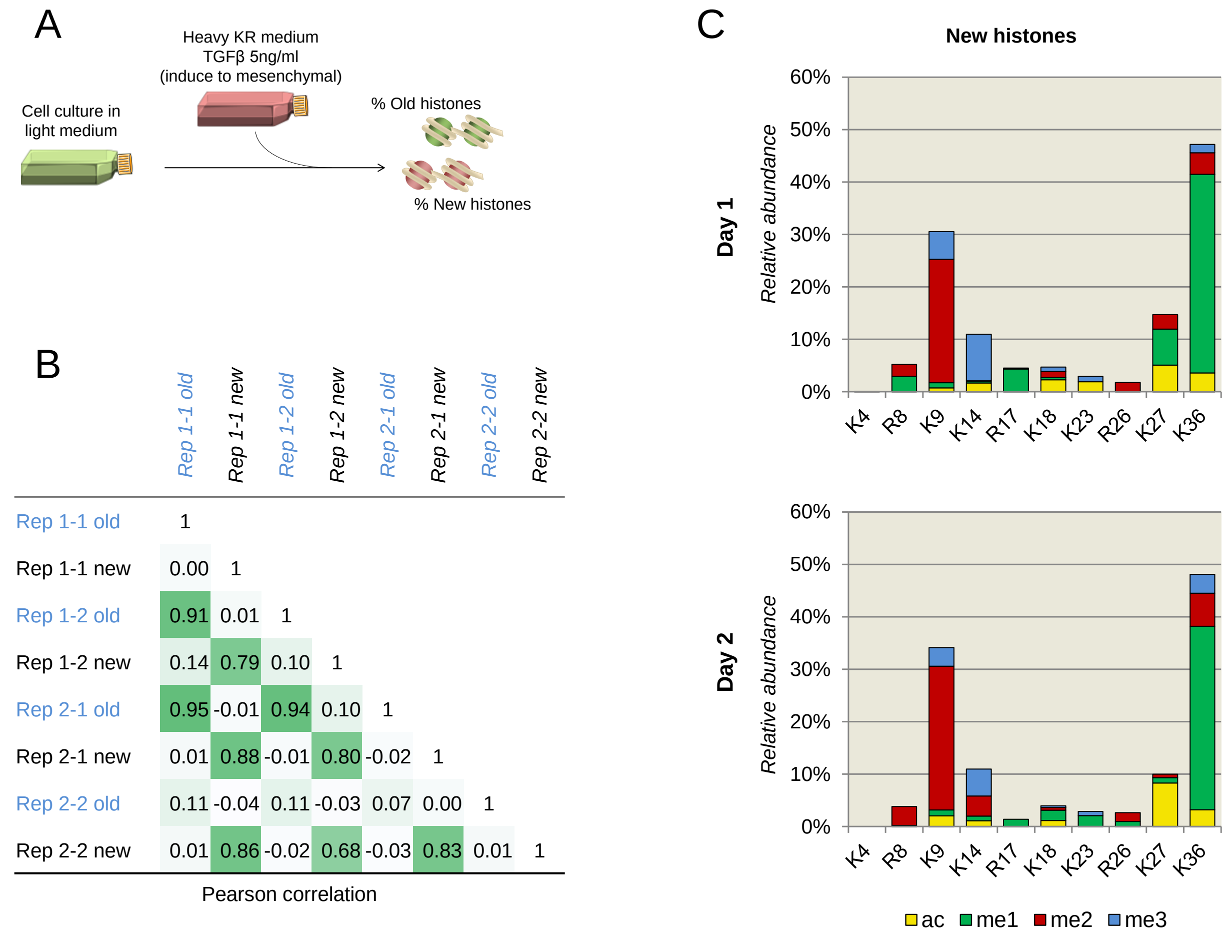

	Rep 1-1 old	Rep 1-1 new	Rep 1-2 old	Rep 1-2 new	Rep 2-1 old	Rep 2-1 new	Rep 2-2 old	Rep 2-2 new
Rep 1-1 old	1							
Rep 1-1 new	0.00	1						
Rep 1-2 old	0.91	0.01	1					
Rep 1-2 new	0.14	0.79	0.10	1				
Rep 2-1 old	0.95	-0.01	0.94	0.10	1			
Rep 2-1 new	0.01	0.88	-0.01	0.80	-0.02	1		
Rep 2-2 old	0.11	-0.04	0.11	-0.03	0.07	0.00	1	
Rep 2-2 new	0.01	0.86	-0.02	0.68	-0.03	0.83	0.01	1

Figure S1: **Analysis of EMT cells labeled using heavy lysine/arginine (heavy KR) during mesenchymal transition.** (A) Workflow for metabolic labeling of EMT cells at the histone sequence level (heavy KR). (B) Pearson correlation values between technical and biological replicates (rep 2-1 implies second biological, first technical), differentiated between combinatorial PTMs quantified on unlabeled histone tails (old histones) and heavy labeled histone tails (new). (C) Bar plot representing the relative abundance of single histone PTMs on heavy labeled histone tails at day 1 (top) and day 2 (bottom). Unlabeled histone tails were excessively low abundant to provide a confident quantification of single histone PTMs.

Figure S2

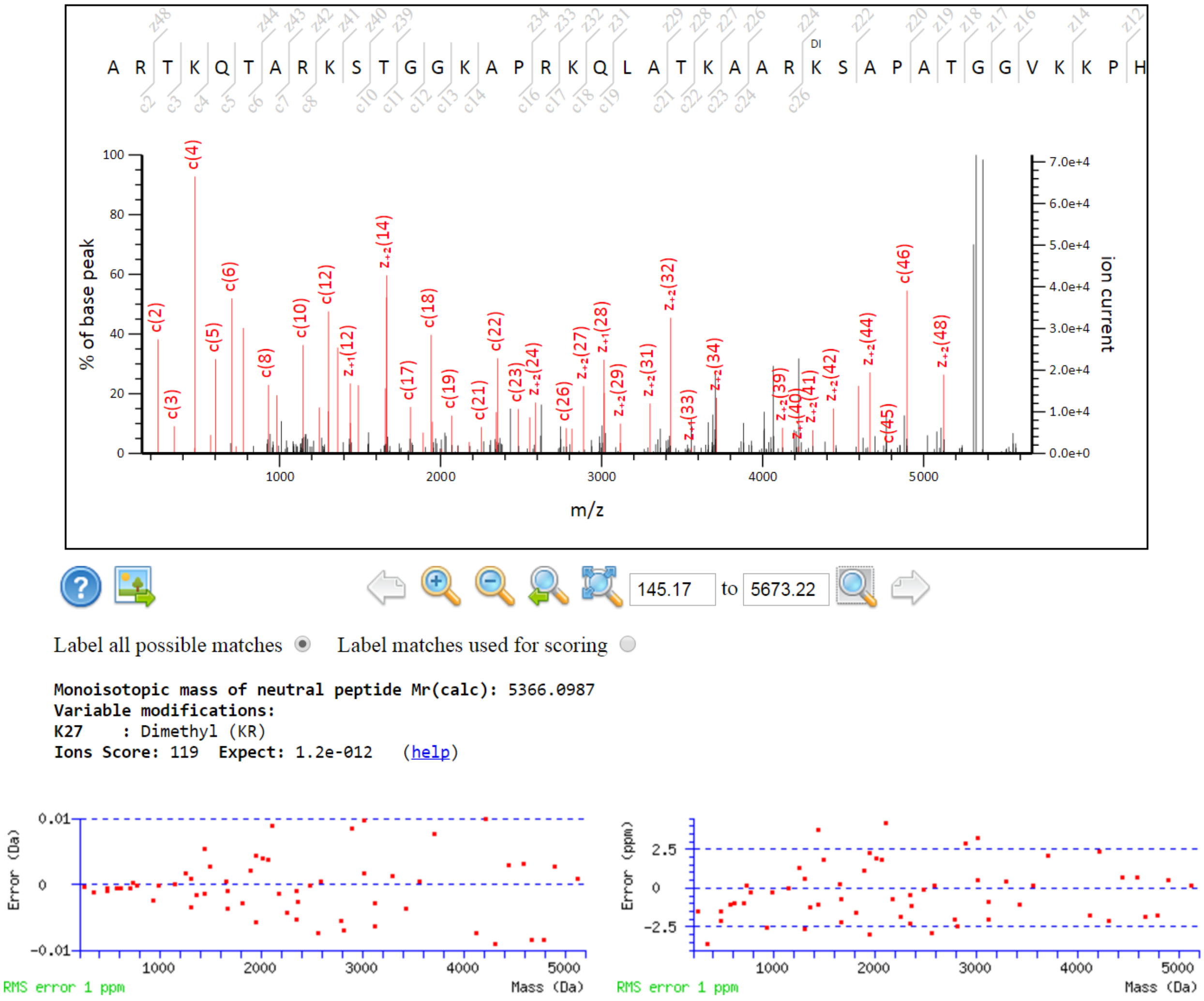

Figure S2: **Annotated MS/MS spectrum of the histone H3 N-terminal tail modified as K27me2.** From the top to bottom, (i) annotated sequence with highlights of identified fragments and modified site, (ii) annotated spectrum, (iii) observed mass, identified modification and Mascot (Matrix Science) identification scores, (iv) distribution of the fragment mass error, expressed in Da (left) and ppm (right).

Figure S3

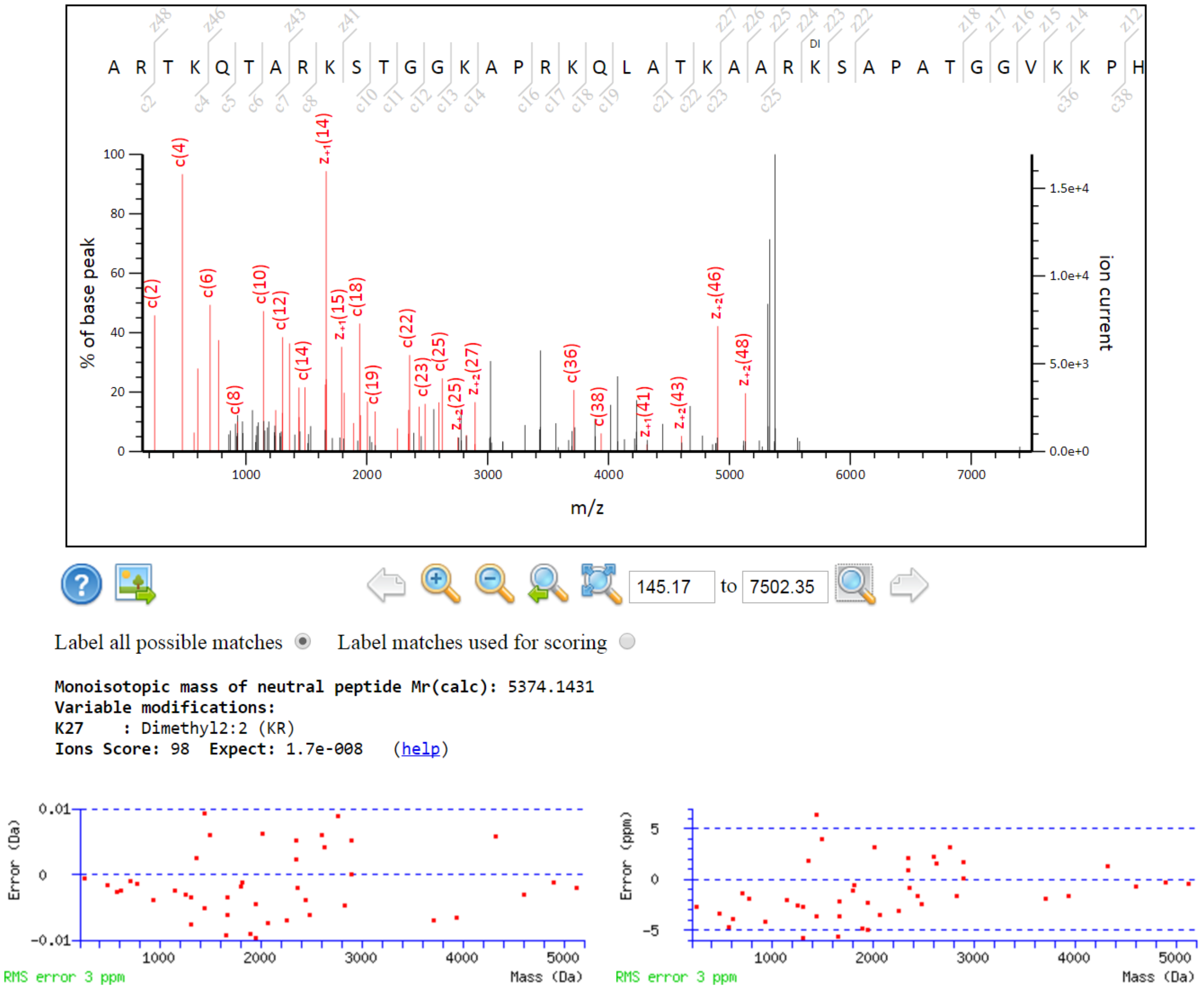

Figure S3: **Annotated MS/MS spectrum of the histone H3 N-terminal tail modified as K27me2:2.** From the top to bottom, (i) annotated sequence with highlights of identified fragments and modified site, (ii) annotated spectrum, (iii) observed mass, identified modification and Mascot (Matrix Science) identification scores, (iv) distribution of the fragment mass error, expressed in Da (left) and ppm (right).

Figure S4

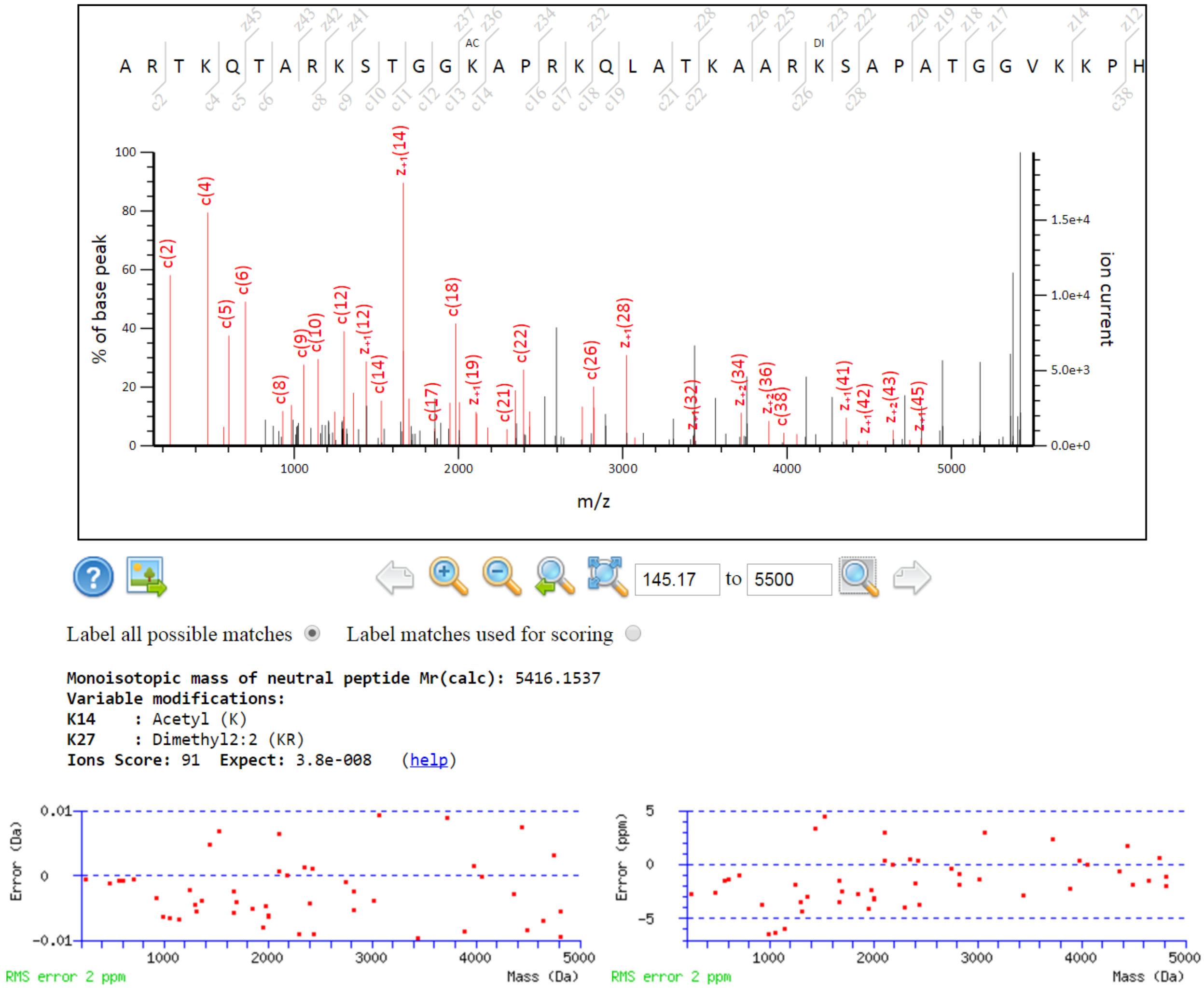

Figure S4: **Annotated MS/MS spectrum of the histone H3 N-terminal tail modified as K14acK27me2:2.** From the top to bottom, (i) annotated sequence with highlights of identified fragments and modified site, (ii) annotated spectrum, (iii) observed mass, identified modification and Mascot (Matrix Science) identification scores, (iv) distribution of the fragment mass error, expressed in Da (left) and ppm (right).

Figure S5

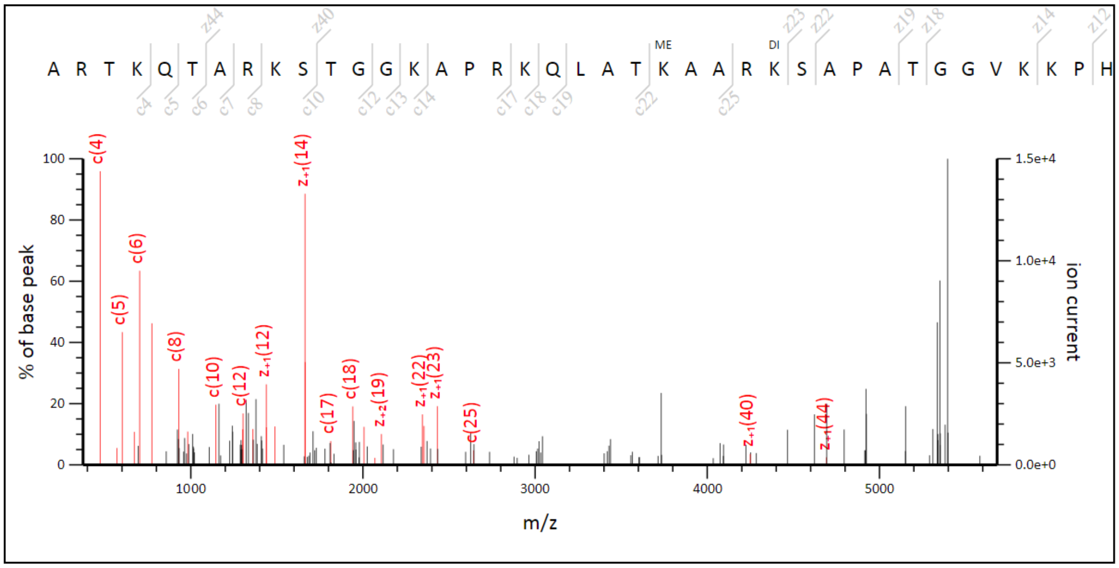

374.31 to 5683.19

Label all possible matches   Label matches used for scoring

```
Monoisotopic mass of neutral peptide Mr(calc): 5392.1809
Variable modifications:
K23    : Methyl1:1 (KR)
K27    : Dimethyl2:2 (KR)
Ions Score: 26  Expect: 0.26   (help)
```

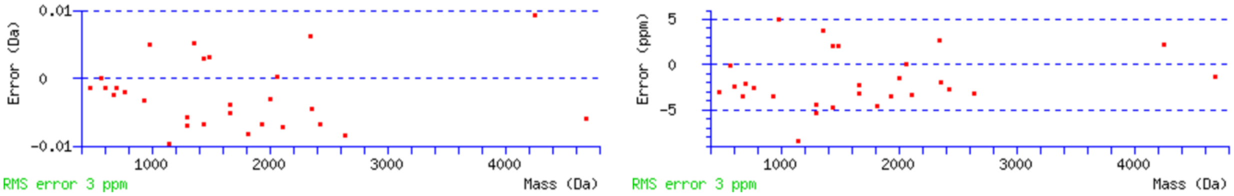

Figure S5: **Annotated MS/MS spectrum of the histone H3 N-terminal tail modified as K23me1:1K27me2:2.** From the top to bottom, (i) annotated sequence with highlights of identified fragments and modified site, (ii) annotated spectrum, (iii) observed mass, identified modification and Mascot (Matrix Science) identification scores, (iv) distribution of the fragment mass error, expressed in Da (left) and ppm (right).

Figure S6

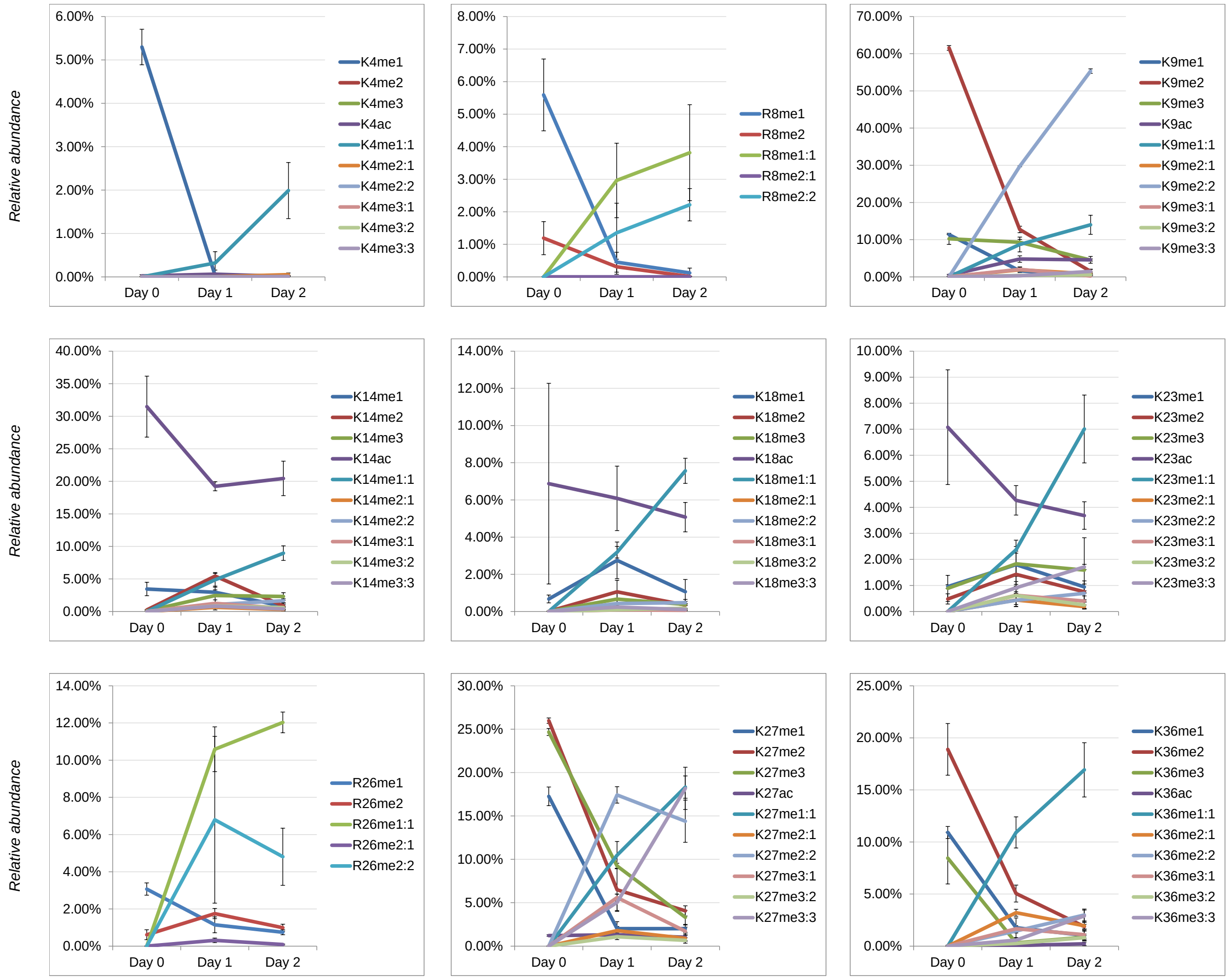

Figure S6: **Relative abundance of all quantified methylations during EMT stimulation combined with heavy methyl labeling.** Each plot represents the trend line of differently methylated PTM sites. Error bar represents standard deviation between three biological replicates (two for day 0).

Figure S7

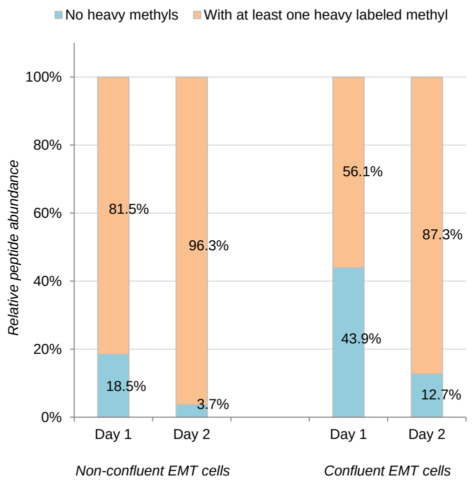

Figure S7: **Sum of all quantitative values of peptides identified in the EMT cell lines incubated with heavy methionine during transition.** The relative abundances represent the total quantification of peptides carrying no heavy labeled methylations (in blue) vs peptides carrying at least one heavy labeled methylation (in orange). Evidently, cells growing in a non-confluent state acquire heavy methylation faster than confluent cells, which have reduced growth rate.

Table S1: **Bottom-up MS quantification of epithelial cells grown in light and heavy KR medium and mixed 1:1.** Relative abundance of bottom-up sized histone peptides labeled with light and heavy KR. The first columns display the relative abundance of the same peptide in unlabeled (light) versus heavy KR labeled form. The columns H/L ratio represent the ratio of the peptides between heavy and light. On the top right, average of H/L ratios across all detected peptides and respective standard deviation. On the bottom right, correlation analysis and significance estimated using the t-test.

Table S2: **Quantification of middle-down sized polypeptides during EMT.** Labeling is illustrated in figure S1A; briefly, epithelial cells were plated into heavy KR media during mesenchymal transition. Unlabeled and heavy KR labeled histone tails are defined as old and new, respectively. Two biological replicates were performed for each experiment. On the right, correlation analysis and significance estimated using the t-test.

Table S3: **Relative abundance of combinatorial PTMs in the EMT experiment including heavy methylation labeling.** Labeling is illustrated in figure 2B; briefly, epithelial cells were plated into heavy methionine media during mesenchymal transition. Biological replicates were performed for the not confluent experiment. On the right, deconvoluted abundance of single histone marks. On the further right, alignment with results obtained from the bottom-up analysis on the same samples. Confluent experiment results are listed in the second sheet.
